# Extra-nuclear effects of estrogen on cortical bone in males require ERαAF-1

**DOI:** 10.1530/JME-16-0209

**Published:** 2017-01-25

**Authors:** H H Farman, J Wu, K L Gustafsson, S H Windahl, S H Kim, J A Katzenellenbogen, C Ohlsson, M K Lagerquist

**Affiliations:** 1 Centre for Bone and Arthritis ResearchInstitute of Medicine, Sahlgrenska Academy, University of Gothenburg, Gothenburg, Sweden; 2 Department of ChemistryUniversity of Illinois, Urbana, Illinois, USA

**Keywords:** estrogen, estrogen dendrimer conjugate, male skeleton, cortical bone

## Abstract

Estradiol (E2) signaling via estrogen receptor alpha (ERα) is important for the male skeleton as demonstrated by ERα inactivation in both mice and man. ERα mediates estrogenic effects not only by translocating to the nucleus and affecting gene transcription but also by extra-nuclear actions e.g., triggering cytoplasmic signaling cascades. ERα contains various domains, and the role of activation function 1 (ERαAF-1) is known to be tissue specific. The aim of this study was to determine the importance of extra-nuclear estrogen effects for the skeleton in males and to determine the role of ERαAF-1 for mediating these effects. Five-month-old male wild-type (WT) and ERαAF-1-inactivated (ERαAF-1^0^) mice were orchidectomized and treated with equimolar doses of 17β-estradiol (E2) or an estrogen dendrimer conjugate (EDC), which is incapable of entering the nucleus and thereby only initiates extra-nuclear ER actions or their corresponding vehicles for 3.5 weeks. As expected, E2 treatment increased cortical thickness and trabecular bone volume per total volume (BV/TV) in WT males. EDC treatment increased cortical thickness in WT males, whereas no effect was detected in trabecular bone. In ERαAF-1^0^ males, E2 treatment increased cortical thickness, but did not affect trabecular bone. Interestingly, the effect of EDC on cortical bone was abolished in ERαAF-1^0^ mice. In conclusion, extra-nuclear estrogen signaling affects cortical bone mass in males, and this effect is dependent on a functional ERαAF-1. Increased knowledge regarding estrogen signaling mechanisms in the regulation of the male skeleton may aid the development of new treatment options for male osteoporosis.

## Introduction

Estrogens have potent effects in several organ systems in the body, including the skeleton. Decline in estrogen levels in women at menopause and after ovariectomy of female rodents leads to decreased bone mass and treatment with estrogen prevents this bone loss as shown both in human and experimental studies (Lindsay *et al.* 1976, Lindberg *et al.* 2002*b*). Thus, the importance of estrogen for bone health in females is well established. In addition, the importance of estrogens for male skeletal health is being increasingly recognized, and many events during male bone development and adult male bone maintenance are now considered regulated by estrogens (Vanderschueren *et al.* 2014). Estrogen levels in older men have been shown to be associated with bone mass and to predict fracture risk (Slemenda *et al.* 1997, Mellstrom *et al.* 2008), and male age-related bone loss is proposed to be caused mainly by estrogen deficiency (Khosla 2013). Thus, knowledge regarding the mechanisms whereby estrogen affects bone mass in males is important to understand and be able to prevent bone loss in an ageing male population.

The effects of estrogens on the skeleton are mediated via estrogen receptors (ERs), and we and others have demonstrated that ERα mediates the main part of the protective effects of estrogen on bone tissue in both females and males (Vidal *et al.* 2000, Lindberg *et al.* 2002*b*, Sims *et al.* 2003). ERs can exert effects via different signaling pathways. Genomic, or nuclear, signaling pathways involve (i) classical signaling, where ERs translocate as a dimer into the nucleus after ligand binding and the dimer subsequently functions as a transcription factor regulating genes with an estrogen-responsive element in their regulatory promoter sequence and (ii) non-classical signaling involving protein–protein interactions with other transcription factors and subsequent activation or repression of the transcriptional machinery for genes with other responsive elements. Estrogens can also exert effects via nongenomic signaling, involving extra-nuclear ER binding. These extra-nuclear effects can occur within minutes after ligand exposure and involve regulation of ion fluxes, initiation of phosphorylation cascades and induction of enzyme activities, which themselves can modify cellular behavior or affect nuclear ER activation. Extra-nuclear ER effects have been demonstrated in various tissues *in vivo* including female bone (see review Banerjee *et al.* 2014).

To study extra-nuclear estrogenic effects, synthetic ligands have been synthesized. One of these is an estrogen dendrimer conjugate, EDC, in which 17α-ethynyl-E2 (EE2) is attached to a polyamidoamine (PAMAM) dendrimer. EDC, which due to its positive charge and size is excluded from the nucleus, provides means to separate nuclear from extra-nuclear estrogen signaling effects (Harrington *et al.* 2006). A role for extra-nuclear estrogen effects has been demonstrated using EDC in various organs including neuroprotective effects in rat (Yang *et al.* 2010) and promotion of cardiovascular protection (Chambliss *et al.* 2010). EDC has also been shown to replicate E2 effects on osteoblast and osteoclast apoptosis *in vitro* and was recently shown to prevent cortical bone loss caused by estrogen deficiency in female mice (Almeida *et al.* 2010, Martin-Millan *et al.* 2010, Bartell *et al.* 2013). Interestingly, no effects of EDC on uterus or breast cancer growth have been found (Chambliss *et al.* 2010, Bartell *et al.* 2013).

ERα contains two activation function (AF) domains involved in the transcriptional activity of the receptor, the ligand-independent AF-1 and the ligand-dependent AF-2. The AF-1 domain, found in the N-terminal of the receptor, can bind several coregulatory proteins, but is less well characterized compared to the AF-2 domain. We have previously demonstrated that ERαAF-2 is required for estrogenic responses in all tissues evaluated, whereas the role of ERαAF-1 is tissue specific, in both males and females (Borjesson *et al.* 2011, 2013). In both genders, the AF-1 domain was shown to be indispensable for the estrogenic effect on trabecular bone, whereas an effect on cortical bone mass was still present in mice lacking a functional AF-1 domain (Borjesson *et al.* 2011, 2013). Interestingly, a role of ERαAF-1 for extra-nuclear effects of estrogen has been suggested *in vitro* (Song *et al.* 2002).

We have used the synthetic estrogen ligand EDC, which lacks the ability to initiate nuclear estrogen signaling effects, and mice lacking a functional ERαAF-1 domain, to determine the impact of extra-nuclear estrogen-mediated effects on bone in male mice and to study the importance of ERαAF-1 for these possible extra-nuclear effects.

## Materials and methods

### Animals and treatment

All experimental procedures involving animals were approved by the Local Ethics Committee at the University of Gothenburg. ERαAF-1^0^ mice, expressing a truncated 49 kDa ERα protein that lacks the AF-1 domain and wild-type (WT) littermates were generated as previously described (Billon-Gales *et al.* 2009). At 5 months of age, the mice were orchidectomized under isoflurane anesthesia and implanted with osmotic minipumps (model 1004, Alzet, Cupertino, CA, USA) intraperitoneally. The pumps were prepared to deliver 6 µg/day of estradiol (E2, Sigma), an equimolar concentration of an estrogen dendrimer conjugate (EDC), in which 17α-ethynyl-E2 (EE2) is attached to a polyamidoamine (PAMAM) dendrimer, E2 vehicle (Veh) (15% EtOH, 43% DMSO, 42% PBS) or EDC vehicle (DC) (poly(amido)amine (PAMAM) dendrimer, 26% MeOH, 74% deionized H_2_O). After 3.5 weeks, the experiment was terminated.

### Bone measurements

Cortical bone was analyzed by peripheral quantitative computed tomography (pQCT), using a Stratec pQCT XCT Research M densitometer with software, version 5.4B (Norland, Fort Atkinson, WI, USA) at a resolution of 70 μm, in tibia at a distance distal to the growth plate corresponding to 30% of the bone length, as previously described (Vidal *et al.* 2000). Trabecular bone in lumbar vertebrae L_5_ was analyzed using a high-resolution μCT, 1172 model scanner (Skyscan), as previously described (Moverare-Skrtic *et al.* 2014). Briefly, the vertebral body caudal of the pedicles was selected for analysis within a volume of interest (cortical bone selectively excluded) commencing at a distance of 4.5 μm caudal of the lower end of the pedicles and extending 225 µm in the caudal direction.

### Serum markers of bone remodeling

Serum levels of degradation products of collagen type I c-telopeptides (CTx) and osteocalcin were assessed using ELISAs as markers of bone resorption and bone formation, respectively (RatLaps ELISA kit, Nordic Bioscience Diagnostics, Herlev, Denmark; Mouse Osteocalcin ELISA kit, number 60-1305, Immutopics, Inc).

### Statistical analysis

Student’s *t*-test was used to assess differences between vehicle and respective treatment group. *P* < 0.05 was considered statistically significant.

## Results

To assess the involvement of extra-nuclear estrogen signaling for the male skeleton and to determine the importance of the ERαAF-1 domain for these effects, we studied the skeletal effects in WT and ERαAF-1-inactivated male mice after treatment with E2 and EDC and corresponding vehicles.

### Effects on cortical bone

**Figure 1:**
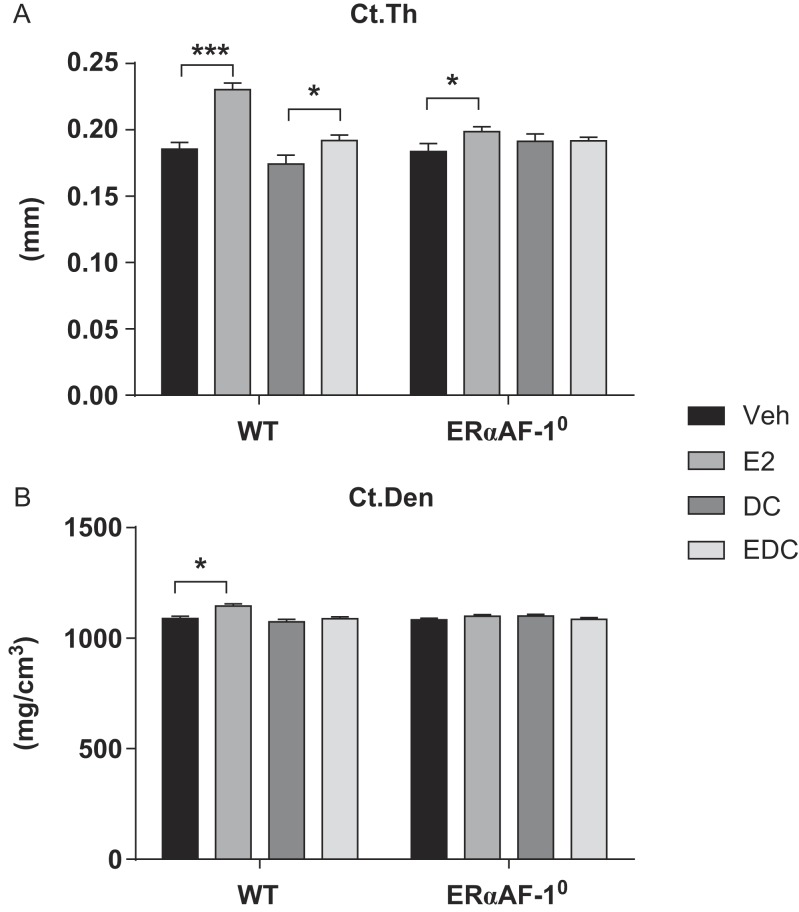
ERαAF-1 is important for the extra-nuclear estrogenic effects on cortical bone in males. Cortical thickness (Ct.Th) (A) and density (Ct.Den) (B) were analyzed in tibia of orchidectomized WT and ERαAF-1^0^ male mice treated with Veh, E2, DC or EDC for 3.5 weeks. *P < 0.05 and ***P < 0.001, Student’s *t* test. Data are presented as mean ± s.e.m. (*n* = 9–11).

Treatment with E2 increased cortical thickness in tibia in both WT (+24%, *P* < 0.001) and ERαAF-1^0^ males (+8%, *P* < 0.05). Thus, part of the estrogenic effects on cortical bone thickness in males is independent of the ERαAF-1 domain (approximately 30%), whereas part of the effect of estrogen requires this domain (Fig. 1A). Interestingly, treatment with EDC resulted in a moderate increase in cortical thickness in WT male mice (+10%, *P* < 0.05) as compared to vehicle treatment, whereas no significant extra-nuclear estrogen effect was detected in mice lacking a functional ERαAF-1 domain (Fig. 1A). As expected, E2 treatment increased cortical bone mineral density (BMD) (+5%, *P* < 0.01) in WT males, but in contrast to what was found when analyzing cortical thickness, no significant treatment effect of E2 was detected in ERαAF-1^0^ mice, and EDC did not affect cortical BMD neither in WT nor in ERαAF-1^0^ male mice (Fig. 1B).

### Effects on trabecular bone

**Figure 2:**
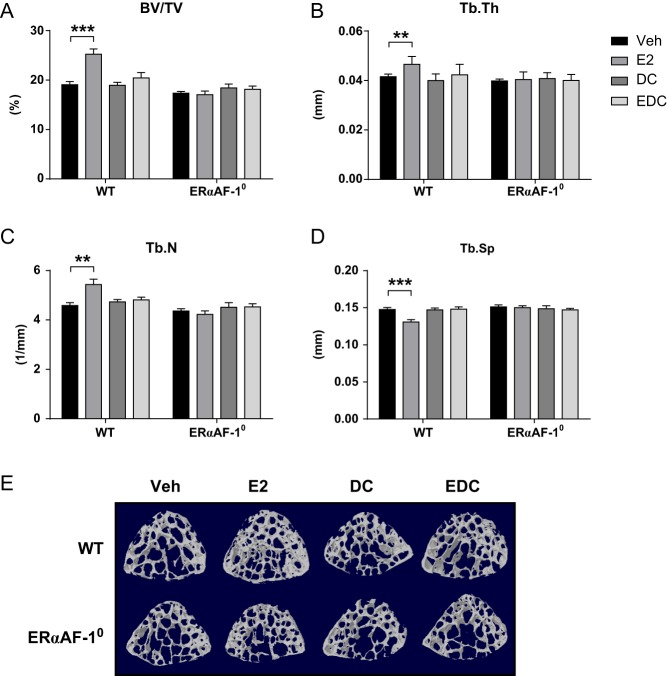
EDC-initiated extra-nuclear signaling does not affect trabecular bone in males. Trabecular bone volume per total volume (BV/TV) (A), thickness (Tb.Th) (B), trabecular number (Tb.N) (C) and trabecular separation (Tb.Sp) (D) in vertebrae (L_5_) of orchidectomized WT and ERαAF-1^0^ male mice treated with Veh, E2, DC or EDC for 3.5 weeks. (E) Representative scans of trabecular bone in vertebrae L_5_. **P < 0.01 and ***P < 0.001, Student’s *t* test. Data are presented as mean ± s.e.m. (*n* = 9–11).

To assess the impact of E2 and EDC treatment on the trabecular bone compartment, the vertebrae (L_5_) was analyzed. E2 treatment significantly increased trabecular bone volume per total volume (BV/TV) (+33%, *P* < 0.001, Fig. 2A) in WT males compared to vehicle treatment. The increase in trabecular bone mass was attributed to increased trabecular thickness (+12%, *P* < 0.01, Fig. 2B) and number (+19%, *P* < 0.01, Fig. 2C) and decreased trabecular separation (−11%, *P* < 0.001, Fig. 2D). Treatment with E2 in ERαAF-1^0^ males did not affect any of the trabecular bone parameters compared to that in vehicle, demonstrating a vital role of the ERαAF-1 domain for estrogenic effects on the trabecular bone compartment (Fig. 2A–E). Furthermore, treatment with EDC did not affect any of the trabecular parameters in neither WT nor ERαAF-1^0^ males, suggesting that extra-nuclear estrogenic effects are not involved in the regulation of the trabecular bone compartment in male mice.

### Effects on bone remodeling serum markers

To assess the effect of E2 and EDC treatment on osteoclast function, degradation products of collagen c-telopeptides (CTx) were measured in serum at the end of the experiment. There was a tendency to decreased CTx levels after E2 treatment in WT males compared to vehicle treatment and treatment with EDC significantly reduced the CTx levels in WT males (Table 1). In males lacking the ERαAF-1 domain, neither E2 nor EDC treatment elicited an effect compared to vehicle treatment (Table 1). Osteocalcin is considered a biomarker for bone formation and was measured in serum at the end of the experiment. No effect of E2 or EDC treatment was detected in WT or ERαAF-1^0^ males (Table 1).

**Table 1:** Serum markers of bone resorption and bone formation after treatment with E2 or EDC in WT and ERαAF-1^0^.

		Veh	E2	DC	EDC
CTx (ng/mL)	WT	51.8 ± 11.8	27.8 ± 1.91#	49.3 ± 8.5	27.5 ± 2.9*
	ERαAF-10	33.2 ± 6.15	27.4 ± 2.5	27.1 ± 2.1	36.5 ± 12.4
Osteocalcin (ng/mL)	WT	86.3 ± 7.6	76.0 ± 6.5	80.4 ± 5.2	78.1 ± 2.1
	ERαAF-10	77.5 ± 4.7	84.0 ± 6.3	77.8 ± 3.9	70.5 ± 3.5

Serum analyses of CTx (bone resorption marker) and osteocalcin (bone formation marker) in orchidectomized WT and ERαAF-1^0^ male mice treated with either estradiol (E2, 6 µg/mouse/day), an equimolar concentration of estrogen dendrimer conjugate (EDC), E2 vehicle (Veh) or EDC vehicle (DC) for 3.5 weeks.

* *P* < 0.05, EDC vs DC, ^#^ *P* = 0.1, E2 vs Veh, Student’s *t* test. Data are presented as mean ± s.e.m. (*n* = 9–11).

## Discussion

Estrogens are crucial for regulation of bone mass in women. However, several studies in both man and mouse demonstrate that estradiol (E2) is also of importance for both growth and adult skeletal maintenance in males (see Manolagas *et al.* 2013, Vanderschueren *et al.* 2014 for review). Life-time risk of osteoporotic fractures after age 50 years may be as high as 20–25% in males, and it is suggested that estrogen deficiency may contribute to this age-related bone loss in men (Khosla 2013, Vanderschueren *et al.* 2014). Thus, to find new treatment options to prevent fractures in men, it would be beneficial to improve our understanding of the bone-specific signaling mechanisms for the skeletal effects of estrogen in males.

The importance of extra-nuclear actions of estrogen for bone mass has recently been shown in female mice (Bartell *et al.* 2013). Using the EDC compound, which provides the means to selectively initiate extra-nuclear estrogen signaling, Bartell and coworkers demonstrated that extra-nuclear estrogen signaling can partly protect against ovariectomy-induced cortical bone resorption, whereas extra-nuclear estrogen signaling in trabecular bone had no effect, suggesting that nuclear-initiated estrogen actions are more important in this bone compartment (Bartell *et al.* 2013). Furthermore, we recently demonstrated that targeting of different domains of ERα results in tissue-specific effects (Borjesson *et al.* 2011, 2013). Male and female mice lacking a functional ERαAF-1 domain did not respond to estrogen in the trabecular bone compartment, whereas there was a clear response to estrogen in the cortical bone compartment, and this ERαAF-1 independency in cortical bone differed somewhat between genders with 2/3 of the estrogenic cortical effect being ERαAF-1 independent in females, whereas approximately 1/3 in males (Borjesson *et al.* 2011, 2013).

As a role of extra-nuclear estrogen signaling was shown recently in female mice, we decided to evaluate the effects of extra-nuclear estrogen actions also in male mice. Furthermore, the ERαAF-1 domain has recently been shown to interact with proteins that are involved in the regulation of extra-nuclear signaling (Jiang *et al.* 2013), and therefore we wanted to characterize the importance of the ERαAF-1 domain for these possible extra-nuclear effects.

**Figure 3:**
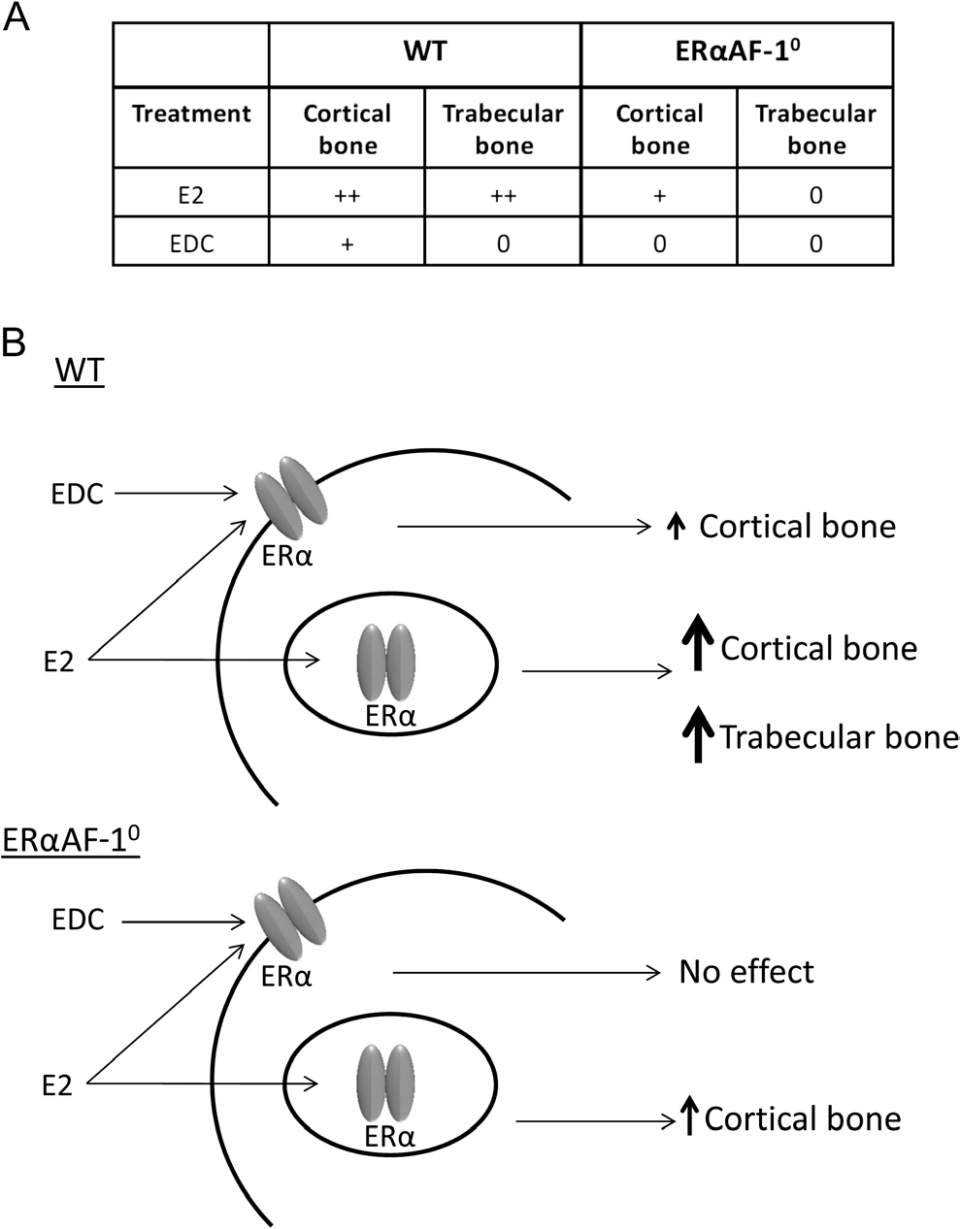
Summary of E2 and EDC effects in WT and ERαAF-1^0^ males. E2 treatment affects both cortical and trabecular bone in WT males and has a moderate effect on cortical, but not trabecular, bone in ERαAF-1^0^ males. EDC treatment only affects cortical bone in WT males, and this effect is abolished in ERαAF-1^0^ mice. (A) ++, normal effect; +, moderate effect; 0, no effect. (B) Schematic presentation of the main proposed effects of EDC and E2 in WT and ERaAF-1^0^ male mice.

E2 treatment has been shown to affect both cortical and trabecular bone in males, and these effects are mediated via ERα, as demonstrated using global ERα knockout mice (Lindberg *et al.* 2002*a*, Moverare *et al.* 2003; Sims *et al.* 2003). In this study, we found clear significant effects on both the trabecular and cortical bone compartments after E2 treatment in WT mice (Fig. 3). However, in a recent study by Ucer and coworkers, E2 treatment resulted in a clear increase in cortical thickness, whereas the effect on trabecular bone was modest (Ucer *et al.* 2016). This difference in impact of E2 treatment on trabecular vs cortical bone compared to our study might depend on differences in treatment duration.

The results in this study showed that EDC, similar as seen in female mice, could increase cortical bone thickness in ORX mice, but had no significant effect on the trabecular bone compartment (Fig. 3). This suggests that extra-nuclear estrogen actions are involved in the positive effect of estrogen treatment on cortical bone in males, whereas the trabecular bone compartment seems to be unaffected by extra-nuclear estrogen effects, at least with the experimental setup used in this study. As previously shown, the trabecular bone of male mice lacking ERαAF-1 did not respond to E2 treatment, whereas the estrogen response in cortical bone was partly independent of ERαAF-1 (Fig. 3). Interestingly, EDC treatment of ERαAF-1^0^ males did not affect the cortical bone, indicating that the effect of extra-nuclear estrogen signaling on cortical bone is dependent on a functional ERαAF-1 in male mice (Fig. 3). Bartell and coworkers suggested that extra-nuclear estrogen actions may have a relatively smaller impact on the trabecular vs the cortical bone compartment (Bartell *et al.* 2013), and we show that this assumption may hold true in both genders. In addition, we saw a strong tendency to decreased CTx after E2 treatment and decreased CTx levels after EDC suggesting that the increased cortical thickness may be due to decreased bone resorption. Tissue for further osteoclast examination was not available, which is a limitation of the study; however, it has previously been shown *in vitro* that EDC treatment increases osteoclast apoptosis (Martin-Millan *et al.* 2010). Thus, extra-nuclear estrogen signaling via ERαAF-1 most likely affects cortical bone via effects on osteoclasts. However, it is possible that these effects on osteoclasts are indirect, initiated by estrogen effects in osteoblasts.

GPR30, a membrane-bound estrogen receptor, has been shown to induce signaling events including intracellular calcium mobilization and cAMP elevation, which are associated with the rapid effects of extra-nuclear estrogen signaling (Filardo *et al.* 2007). GPR30 signaling has been shown to influence the skeleton in both females and males (Windahl *et al.* 2009, Ford *et al.* 2011, Kang *et al.* 2015); however, the likelihood that this receptor is responsible for the effect on cortical bone after treatment with EDC is small since the effect is abolished by deletion of a domain in the ERα receptor.

We have shown that the ERαAF-1 domain is involved in extra-nuclear estrogenic effects on cortical bone in males. Several cytoplasmic adaptor proteins have been shown to interact with membrane/cytoplasmic ERα, including MEMO (Mediator of ERBB2-driven cell MOtility), which can promote extra-nuclear estrogen signaling via interaction with the ERαAF-1 domain (Jiang *et al.* 2013), and MNAR (Modulator of Nongenomic Action of estrogen Receptor) (Fox *et al.* 2009). Thus, a possible mechanism behind the ERαAF-1-dependent extra-nuclear effect on cortical bone may involve interactions of ERα with proteins such as MEMO and MNAR. The effects of estrogen on cortical bone result from both nuclear and extra-nuclear estrogen actions, and cross-talk between these two pathways have been suggested to be of importance in other contexts (Madak-Erdogan *et al.* 2008). Therefore, another possible explanation for the dependency of the ERαAF-1 domain for extra-nuclear estrogen signaling effects on cortical bone is that ERα-AF1 has an essential role in this cross-talk; however, these suggestions are still to be verified.

In conclusion, our results indicate that extra-nuclear estrogen signaling is involved in the effects of estrogen on cortical bone in males and that ERαAF-1 is crucial for this effect. The results of this study highlight the vital role of estrogen signaling for the male skeleton and lead to increased knowledge of the importance of different estrogen signaling pathways in the regulation of bone, which may aid the development of new treatment options for male osteoporosis.

## Declaration of interest

The authors declare that there is no conflict of interest that could be perceived as prejudicing the impartiality of the research reported.

## Funding

Support of this research from the National Institutes of Health (PHS 5R01DK015556), the Swedish Research Council (521-2012-1643), the Swedish Foundation for Strategic Research, the ALF/LUA research grant from the Sahlgrenska University Hospital (ALFGBG-428402), the Lundberg Foundation (Dnr.424), the Torsten (M55/13) and Ragnar (M133/12) Söderberg’s Foundations and the Novo Nordisk Foundation (NNF15Obib16318) is gratefully acknowledged.

## Author contribution statement

H H F, C O and M K L conducted the study design. K L G, M K L, H H F, J W and S H W were responsible for acquisition of data and H H F, M K L, C O, S H K and J A K performed the analysis and interpretation of data. M K L, H H F and C O wrote the main manuscript text and H H F and M K L prepared the figures. All authors reviewed the manuscript.
